# Social Stress-Related Epigenetic Changes Associated With Increased Heart Rate Variability in Infants

**DOI:** 10.3389/fnbeh.2019.00294

**Published:** 2020-01-15

**Authors:** Ghazal Aghagoli, Elisabeth Conradt, James F. Padbury, Stephen J. Sheinkopf, Hasmik Tokadjian, Lynne M. Dansereau, Edward Z. Tronick, Carmen J. Marsit, Barry M. Lester

**Affiliations:** ^1^Brown Center for the Study of Children at Risk, Brown University, Providence, RI, United States; ^2^Department of Neuroscience, Brown University, Providence, RI, United States; ^3^Departments of Psychology, Pediatrics, and Obstetrics and Gynecology, University of Utah, Salt Lake City, UT, United States; ^4^Warren Alpert Medical School, Brown University, Providence, RI, United States; ^5^Department of Pediatrics, Women & Infants Hospital of Rhode Island, Providence, RI, United States; ^6^Department of Psychology, University of Massachusetts Boston, Boston, MA, United States; ^7^Department of Environmental Health, Emory University, Atlanta, GA, United States

**Keywords:** early life stress, epigenetics, heart rate variability, autonomic system reactivity, mother-infant interaction

## Abstract

Early life stress can result in persistent alterations of an individual’s stress regulation through epigenetic modifications. Epigenetic alteration of the *NR3C1* gene is associated with changes in the stress response system during infancy as measured by cortisol reactivity. Although autonomic nervous system (ANS) reactivity is a key component of the stress response, we have a limited understanding of the effects of *NR3C1* DNA methylation on ANS reactivity. To examine this relation, ANS stress responses of term, 4–5-month-old healthy infants were elicited using the face-to-face still-face paradigm, which involved five, 2-min episodes. Two of these episodes were the “still-face” in which the mother was non-responsive to her infant. EKG was acquired continuously and analyzed in 30 s-intervals. Cheek swabs were collected, and DNA was extracted from buccal cells. Respiratory sinus arrhythmia (RSA) was measured as heart rate variability (HRV). Mean HRV was calculated for each 30-s “face to face” episode. DNA methylation of *NR3C1* was calculated using bisulfite pyrosequencing. Percent DNA methylation was computed for each of the 13 *NR3C1* CpG sites. The relations between mean HRV for each “face to face” episode and percent DNA methylation was examined averaged over CpG sites 1–6 and 7–13 and at each individual CpG site. Higher HRV at baseline, first reunion, and second still-face was related to greater methylation of *NR3C1* CpG sites 1–6. Higher HRV at the second reunion was related to greater methylation of *NR3C1* CpG sites 12 and 13. These data provide evidence that increased methylation of *NR3C1* at CpG sites 12 and 13 are associated with increased activation of parasympathetic pathways as represented by increased HRV.

## Introduction

Early adversity can result in persistent alterations of an individual’s physiological stress response due, in part, to epigenetic changes. There is growing evidence that prenatal and perinatal exposure to early life stress or environmental adversity may influence gene expression patterns through DNA methylation in which the addition of a methyl group to CpG sites in the promoter region of a gene, down or upregulate gene expression (Liu et al., 1997; Weaver et al., 2004, 2007; Meaney, 2010).

The *NR3C1* gene codes for the glucocorticoid receptor (GR), which primarily functions as a gene transcription regulator. GR either upregulates the expression of anti-inflammatory proteins or represses the expression of pro-inflammatory proteins in the cell. Methylation of the *NR3C1* promoter region is associated with increased stress response in infancy as measured by cortisol reactivity (Oberlander et al., 2008; Tyrka et al., 2012; Conradt et al., 2013).

Studies that examine the relation between *NR3C1* methylation and physiological stress responses typically focus on the HPA axis and cortisol reactivity, a stress response has a different mechanism from the autonomic nervous system (ANS) response involving sympathetic and parasympathetic activation. In previous work, we found that breastfeeding as a proxy for maternal care was associated with decreased DNA methylation of *NR3C1* and decreased cortisol reactivity in 4–5-month-old infants (Lester et al., 2018). The ANS also plays a significant role in generating adaptive responses to physiological and/or psychological stress. The presence of a threat results in the activation of the ANS which activates the sympathetic branch, resulting in increased arousal and energy mobilization, the so-called “fight or flight” response. The ANS also activates the parasympathetic branch through the stimulation of the nucleus ambiguous and dorsal motor nucleus of the vagus nerve (Ulrich-Lai and Herman, 2009) to act on the sinoatrial (SA) node and limit the duration of arousal state (Kolman et al., 1975). Therefore, the central autonomic network (CAN) controls sympathoexcitatory subcortical threat circuits through inhibition (Benarroch, 1993; Amat et al., 2005; Thayer, 2006). Heart rate variability (HRV) is a widely used peripheral measure of ANS activity and represents the balance between sympathetic and parasympathetic branches. One measure of HRV is respiratory sinus arrhythmia (RSA), which measures variability in heart rate that coincides with breathing, reflecting parasympathetic control (Bernston et al., 1997). Prolonged exposure to stress is associated with autonomic imbalance; a hyperactive sympathetic system and a hypoactive parasympathetic system (Thayer, 2006). Under prolonged conditions of threat and uncertainty, the CAN is hypoactive, disinhibiting the sympathoexcitatory circuits, resulting in a sympathetic response and energy depletion. The lack of this inhibitory mechanism is reflected in deficits such as poor habituation to novel neutral stimuli, a pre-attentive bias for threat information, and poor regulation (Thayer, 2006).

We studied RSA in 4–5-month-old infants during a face-to-face still-face paradigm that included an episode in which the infant was exposed to a social stressor (Tronick et al., 1978) to determine the relation between *NR3C1* methylation and ANS reactivity. We hypothesized that increased methylation of the *NR3C1* promoter region in infants was associated with increased RSA. To our knowledge this is the first study relating *NR3C1* epigenetic alterations to the ANS in the form of HRV.

## Materials and Methods

### Participants

Mothers and their infants were recruited from an existing cohort of infants (Rhode Island Child Health Study, Marsit et al., 2012) at Women and Infants Hospital of Rhode Island (WIHRI; Conradt et al., 2016b; Lester et al., 2018). The study was approved by the WIHRI Institutional Review Board. All mothers gave written informed consent.

Term infants born appropriate for gestational age (AGA), based on birth weight, gestational age, and calculations from the Fenton Growth Chart for Preterm Infants (Fenton and Kim, 2013) were identified and approached for participation. The exclusion criteria were maternal age below 18 or above 40, non-singleton birth, a life-threatening medical complication for the mother, and congenital or chromosomal abnormality of the infant, and a language other than English as their primary language.

### Stress Reactivity

Stress reactivity was elicited from the infant using a face-to-face still-face paradigm (Tronick et al., 1978), a mother-infant interaction paradigm during which the mother is non-responsive to her infant. During the first episode, the mother is instructed to interact with her infant normally as she would at home for 2 min while the infant sits in an infant seat across from the mother. During the “still-face” episode, the mother is instructed to maintain a blank “poker” face and not to smile and interact with her infant for 2 min. It is typical that infants show increases in crying and decreases in positive affect in response to the “still face.” During the third episode, the reunion, the mother and infant play together as they normally would for 2 min. The modification by Haley and Stansbury (2003) includes an additional still-face and reunion episode. The “still-face” is a stressor and has been reliably shown to elicit stress response (Haley and Stansbury, 2003; Lester et al., 2018). EKG was acquired continuously during the procedure, and cardiac parameters were summarized in 30-s blocks, as described below.

### EKG Acquisition and HRV Analysis

EKG was acquired using BIOPAC EKG bio amplifier (MP150CE, BIOPAC Systems, Inc., Goleta, CA, USA) at a sampling rate of 1 kHz using a three electrode placement on infants chest and abdomen. The resulting EKG waveform time series was synchronized to the video recording of the face-to-face still—face procedure. Processing of the EKG was performed using MindWare (MindWare Technologies Limited, 2014) and included inspection for artifacts and missed or spurious beats (R-waves). This data processing was performed in 30-s windows aligned with the start of each 2-min episode in the face-to-face still-face procedure (Patriquin et al., 2013; Conradt et al., 2016a; Sheinkopf et al., 2007). Heart rate (beats per minute) and HRV were extracted from each processed 30-s analysis window and then averaged across the analysis windows for each of the face-to-face still-face episodes. We used RSA to measure HRV, calculated as the amplitude of periodicity in HR within the high-frequency band (0.24–1.04 Hz; Bar-Haim et al., 2000). Mean RSA was calculated by averaging the RSA values obtained within each episode (five RSA data windows at baseline, and four RSA data windows at all other episodes). The Reactivity was calculated by taking the mean difference between HRV during the still-face and reunion episodes (both times).

### Buccal Sample Collection, DNA, and Bisulfite Modification

Buccal-derived DNA was collected from cheek swab samples following the face to face still-face paradigm using the Oragene-DNA assisted collection system. The swabs were then placed in a collection tube and sealed, releasing a stabilizing solution into the collected sample to allow for processing of the sample at a later period. DNA was isolated from the collection tubes following the Oragene methods. Purified DNA was quantified using an ND-1000 spectrophotometer (Nanodrop, Wilmington, DE, USA), and DNA samples (500 ng) were bisulfite modified using the EZ DNA Methylation Kit and stored at −20°C.

### Bisulfite Pyrosequencing DNA Methylation Analysis *NR3C1*

Pyrosequencing, which allows for quantitative assessment of DNA methylation in short sequence regions, was performed on PCR product amplified from bisulfite-modified DNA as described previously (Conradt et al., 2013).

The primers for amplification were forward: 5′-TTT TTT TTT TGA AGT TTT TTT A-3′ and reverse: 5′-Biotin-CCC CCA ACT CCC CAA AAA-3′. The first sequencing primer was designed to sequence the first 5 CpG sites (5′-GAG TGG GTT TGG AGT-3′), and the second sequencing primer was designed to sequence the following 8 CpG sites (5′-AGA AAA GAA TTG GAG AAA TT-3′) for a total of 13 sites sequenced from the *NR3C1* gene.

The percent methylation at each CpG site was quantified using the Pyro Q-CpG Software, version 1.0.11 (Qiagen, Germantown, MD, USA). Bisulfite conversion controls were included in each sequencing read. In order for the sample’s methylation extent to be called, the bisulfite conversion rate must be >93%, and for all samples examined the conversion rate was >95%. All assays were performed in triplicate on the same bisulfite converted DNA template on all samples, and if any of the repeats differed by >10% those assays on that sample were repeated. To prevent batch effects from bisulfite treatments interfering with the analysis, samples were randomized across batches.

### Missing Data

There were 125 terms, AGA, healthy 4-month-old infants with complete *NR3C1* methylation data. Of these, 23 infants also had HRV data at baseline but did not have complete HRV data for all episodes.

### Statistical Analysis

Statistical analysis was performed using SPSS Statistics 17.0. The relations between *NR3C1* promoter region methylation and HRV were examined using Pearson correlations. We examined mean percent DNA methylation averaged over blocks of GpG sites (1–6 and 7–13) and at individual CpG sites. Averaging across blocks of sites is justified when the distribution of mean percent of DNA methylation shows distinct patterns for sites closer to the promoter region (1–6) compared with sites more distal to the promoter region (sites 7–13) which is often the case for *NR3C1* (Lester et al., 2018) and affords the opportunity to identify more global findings. However, this does not allow for consideration of the role of transcription factors that are specific to individual CpG sites. Accordingly, we also computed correlations between the mean DNA methylation at each CpG site (ranging from 1 to 13) in the *NR3C1* gene and mean RSA at each episode of the still-face procedure.

Linear regression was used for the analysis of mean *NR3C1* DNA methylation, mean RSA at each episode and RSA reactivity. We tested the main effects of HRV on DNA methylation of NR3C1 CpG sites 1–6, 7–13, and individual sites. We examined covariates that may be related to DNA methylation of *NR3C1* including gestational age, birth weight, ethnicity, and gender.

Multiple testing was accounted for using PROC MULTEST in SAS with the FDR option. These option requests *p*-values that are adjusted to control the “false discovery rate” described by Benjamini and Hochberg (1995). As is standard in the epigenetic literature, we chose a *q* = 0.10.

## Results

Maternal and infant characteristics are included in Table 1. Most of the participants were Caucasian (77%) and the average maternal age was 31.8 (range = 18–38). The average age of the infants was 17.9 weeks (range = 15.7–24.0). There were no differences in any of the maternal and infant characteristics between the 23 subjects included and the 102 subjects without HRV data that were excluded from this study. None of the maternal or infant characteristics were correlated with DNA methylation or HRV.

**Table 1:** Maternal and infant characteristics (*n* = 23).

Demographic variable	% or Mean (SD)
**Maternal**	
Employment	
Full-time work	47.8%
Part-time work	8.7%
Unemployed	8.7%
Household income	
0–24,999	21.7%
25,000–49,999	8.7%
≥50,000	69.6%
Race	
Caucasian	78.3%
African american	8.7%
Asian	4.3%
Native american	4.3%
“Other”	4.3%
Ethnicity	
Hispanic	21.7%
Age	31.3 (4.9)
Education ≥ high school	91.3%
**Infant**	
Gender (Female)	43.5%
Age (weeks)	17.9 (2.1)
Birthweight (grams)	3,463 (666)
Gestational age (weeks)	39.2 (1.2)

Consistent with prior studies, HRV was lower during the still-face episode than during baseline (play) and reunion (Bazhenova et al., 2001; McCormick et al., 2018) Average HRV during the five episodes in the face-to-face still-face paradigm can be seen in Table 2.

**Table 2:** Heart rate variability (HRV) at each of the five episodes of the face-to-face still-face paradigm [HRV is represented by mean respiratory sinus arrhythmia (RSA) values].

Mean RSA	Mean (SD)
Baseline	3.33 (0.70)
First still-face	2.91 (1.03)
First reunion	3.40 (0.80)
Second still-face	3.02 (1.16)
Second reunion	3.11 (0.85)
First reactivity	0.47 (0.73)
Second reactivity	0.25 (0.82)

Table 3 includes the correlations between HRV and *NR3C1* methylation at each CpG site during the face to face still face episodes. Higher HRV in infants was related to greater DNA methylation levels of *NR3C1* CpG sites 1–6 at baseline (*r* = 0.511, *p* = 0.013, *q* = 0.037), first reunion (*r* = 0.491, *p* = 0.020, *q* = 0.037), and second still-face (*r* = 0.487, *p* = 0.022, *q* = 0.037).

**Table 3:** Correlations between HRV at each face to face still-face episode and NR3C1 methylation at CpG sites 1–13.

	Baseline (*n* = 23)		First Still-Face (*n* = 21)		First Reunion (*n* = 22)		Second Still-Face (*n* = 22)		Second Reunion (*n* = 20)		Reactivity 1 (*n* = 21)		Reactivity 2 (*n* = 20)	
CpG Position	rho	*p*	rho	*p*	rho	*p*	rho	*p*	rho	*p*	rho	*p*	rho	*p*
*NR3C1* CpG Site 1	0.256	0.264	0.065	0.792	0.200	0.398	0.124	0.601	0.483*	0.036	0.159	0.517	0.309	0.198
*NR3C1* CpG Site 2	0.391	0.080	0.108	0.659	0.399	0.081	0.292	0.211	0.461*	0.047	0.254	0.293	0.066	0.790
*NR3C1* CpG Site 3	0.121	0.602	0.291	0.226	0.186	0.432	0.337	0.146	0.052	0.832	−0.206	0.398	−0.329	0.170
*NR3C1* CpG Site 4	−0.158	0.494	0.211	0.386	−0.010	0.968	0.137	0.565	−0.249	0.304	−0.261	0.281	−0.405	0.085
*NR3C1* CpG Site 5	−0.069	0.765	0.264	0.276	0.073	0.761	0.174	0.464	−0.032	0.896	−0.251	0.300	−0.292	0.225
*NR3C1* CpG Site 6	0.242	0.0291	0.268	0.267	0.214	0.365	0.204	0.387	0.214	0.378	−0.107	0.664	−0.066	0.787
*NR3C1* CpG Site 7	0.421	0.057	0.338	0.157	0.355	0.125	0.286	0.222	0.431	0.066	−0.113	0.644	0.073	0.767
*NR3C1* CpG Site 8	0.283	0.214	0.349	0.143	0.224	0.342	0.253	0.282	0.328	0.170	−0.224	0.357	0.019	0.939
*NR3C1* CpG Site 9	0.364	0.105	0.361	0.129	0.294	0.208	0.268	0.254	0.384	0.105	−0.201	0.410	0.040	0.871
*NR3C1* CpG Site 10	0.409	0.066	0.340	0.154	0.334	0.151	0.252	0.283	0.438	0.061	−0.130	0.596	0.116	0.635
*NR3C1* CpG Site 11	0.384	0.086	0.388	0.101	0.381	0.098	0.371	0.089	0.418	0.066	−0.186	0.419	0.094	0.693
*NR3C1* CpG Site 12	0.366	0.086	0.338	0.135	0.288	0.194	0.266	0.231	0.555**	0.011	−0.195	0.396	0.143	0.548
*NR3C1* CpG Site 13	0.342	0.111	0.324	0.153	0.261	0.241	0.247	0.269	0.634**	0.003	−0.200	0.384	0.157	0.510
*NR3C1* CpG Sites 1-6	0.511**	0.013	0.424	0.055	0.491**	0.020	0.487**	0.022	0.215	0.363	−0.043	0.852	−0.154	0.516
*NR3C1* CpG Sites 7-13	0.345	0.107	0.352	0.118	0.298	0.177	0.259	0.245	0.504*	0.023	−0.197	0.391	0.100	0.674

Note: *significant at *p* < 0.05; **significant at *p* < 0.05 and *q* < 0.10.

One during the second reunion, higher HRV was related to greater methylation at *NR3C1* CpG sites 12 (*r* = 0.555, *p* = 0.011, *q* = 0.083) and 13 (*r* = 0.634, *p* = 0.003, *q* = (0.045).

**Figure 1:**
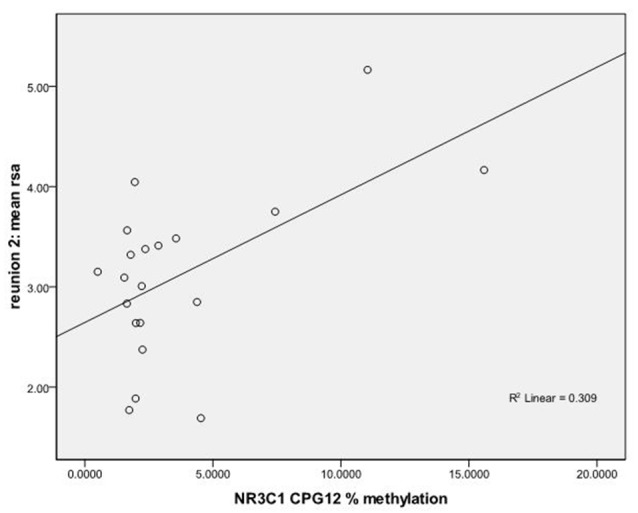
Scatterplot for the correlation between DNA methylation of *NR3C1* CpG 12 and heart rate variability (HRV) at reunion 2 episode. HRV is represented by mean respiratory sinus arrhythmia (RSA) values during reunion 2 episodes (Y-axis). The mean percent of DNA methylation of *NR3C1* at CpG 12 is shown on the X-axis.

**Figure 2:**
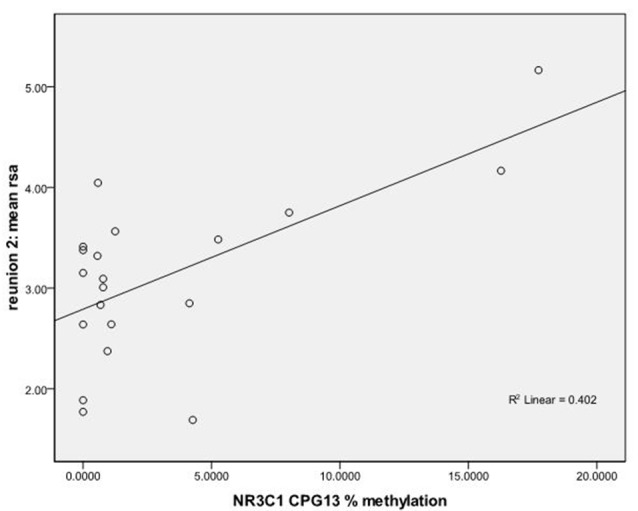
Scatterplot for the correlation between DNA methylation of *NR3C1* CpG 13 and HRV at reunion 2 episode. HRV is represented by mean RSA values during reunion 2 episodes (Y-axis). The mean percent of DNA methylation of *NR3C1* at CpG 13 is shown on the X-axis.

We used regression models to further examine the effects of HRV on DNA methylation of *NR3C1* CpG sites 1–6 and 7–13 and *NR3C1* sites 1–13 individually after adjustment for covariates (Table 4). Higher HRV at baseline was related to greater methylation of *NR3C1* CpG sites 1–6 and 10–11. Higher HRV at first still-face was related to greater methylation of *NR3C1* CpG site 11. Higher HRV at first reunion was related to greater methylation of NR3C1 CpG sites 1–6 and 11. Higher HRV at second still-face was related to greater methylation of *NR3C1* sites 1–6. Higher HRV at the second reunion was related to greater methylation of *NR3C1* CpG sites 7–13 and sites 12, and 13 (Figures 1, 2).

**Table 4:** Regression predicting DNA methylation.

	*B*	*β*	*R*^2^	*p*-value
*NR3C1* CpG Sites 1–6				
Baseline	3.55	0.71	0.48	0.029
First reunion	0.60	0.53	0.45	0.020
Second still-face	0.41	0.56	0.51	0.009
*NR3C1* CpG Sites 7–13				
Second reunion	1.97	0.52	0.40	0.043
*NR3C1* CpG Site 10				
Baseline	2.66	0.62	0.44	0.012
*NR3C1* CpG Site 11				
Baseline	3.55	0.71	0.48	0.004
First still-face	2.34	0.69	0.53	0.003
First reunion	0.60	0.53	0.45	0.018
*NR3C1* CpG Site 12				
Second reunion	2.51	0.56	0.44	0.023
*NR3C1* CpG Site 13				
Second reunion	3.98	0.64	0.54	0.007

## Discussion

We found that methylation of the *NR3C1* promoter region was associated with autonomic HRV responses in 4–5-month-old infants during a mildly stressful parent-infant interaction. These data suggest that percent of methylation is related to physiological recovery in response to the still-face episode. We found that average methylation levels at CpG sites 1–6 were associated with HRV during baseline, the first reunion episode, and the second still faces episode such that greater levels of methylation were associated with greater variability in HR within the frequency band of typical respiration (RSA). The variance in HRV accounted for by percent methylation variance was of medium effect size (Cohen, 1988). We also found that increased average percent methylation of CpG sites 7–13 was correlated with increased variation in HRV during the second reunion episode. The variance in HRV that was accounted for by variation in methylation levels was of large effect size (Cohen, 1988). We found significant associations between methylation levels at CpG sites 12 and 13 and variation in HRV during the second reunion episode. While these significant findings survived a false discovery rate adjustment for multiple tests, our results should be replicated in an independent sample.

To address the issue of differences in DNA methylation between buccal cell samples and brain, using the IMAGE-CpG tool (Braun et al., 2019) we examined the correlation between methylation in buccal and brain samples. Over the entire NR3C1 gene region, this tool demonstrates a correlation of 0.9 between brain and buccal samples. This tool is based on data from the Illumina MethylationEPIC array, which does not interrogate the exact CpGs interrogated *via* pyrosequencing in this study, but using CpG sites from the array surrounding the sites interrogated in this study, there are correlation coefficients ranging from 0.25 to 0.48, suggesting a moderate correlation between buccal and brain samples.

*NR3C1* is, arguably, the most studied candidate gene in human behavioral epigenetic research. There is a long history of research linking differences in *NR3C1* DNA methylation to cortisol reactivity and the HPA response (Liu et al., 1997; Weaver et al., 2004; Oberlander et al., 2008; Romens et al., 2015). Rodent models have revealed that increased maternal licking and grooming (LG) and arched-back nursing led to decreased *Nr3c1* Exon I_7_ DNA methylation (as measured from hippocampal cells; Liu et al., 1997), which in turn resulted in increased expression of GRs and increased activity of HPA axis. This region of the *NR3C1* gene corresponds to exon 1_F_ in the human *NR3C1* gene (Oberlander et al., 2008).

To our knowledge this is the first study relating *NR3C1* epigenetic alterations to the ANS in the form of RSA. The analysis of CpG sites 1–6 vs. CpG sites 7–13 and mean RSA shows that methylation in earlier *NR3C1* exon 1_F_ CpG sites is associated with variability in heart rate during reunion 1; however, with increase in stress or prolonged exposure to stress, as represented by reunion 2 episode, later *NR3C1* exon 1_F_ CpG sites account for variability in heart rate. These sites included CpG sites 12, and 13.

CpG sites 12 and 13 of the *NR3C1* exon 1_F_ gene play an important role in the expression of the *NR3C1* gene. Nerve growth factor-induced protein A (NGFI-A) transcription factor induces *NR3C1* expression by binding to CpG sites 12 and 13 of *NR3C1* exon 1_F_ gene (McGowan et al., 2009). Patch methylation of CpG sites 12 and 13 reduce the binding of NGFI-A transcription factor and reduces transcriptional activation through the *NR3C1* exon 1_F_ promoter region (McGowan et al., 2009). Therefore, increased methylation at CpG sites 12 and 13 results in decreased expression of GRs. Therefore, we would expect that in healthy term infants, an increased response to stress during the still-face episode is followed by increased regulation of that stress during the reunion episode, especially at reunion 2 because the infant faces and thus needs to regulate more stress.

In previous work with this cohort, we found that maternal care impacts the epigenome through behavioral programming which, in turn, alters HPA stress reactivity (Lester et al., 2018; Terell et al., 2019) an observation well documented in rodents (Liu et al., 1997; Weaver et al., 2004, 2007; Meaney, 2010). The current study provides parallel translational evidence and could suggest a mechanistic link for the role of the ANS in the programming of long term stress response systems.

### Limitations of This Study

First, although the relations between *NR3C1* methylation and HRV are consistent with more mechanistic findings in animal models, the results are correlational and we cannot infer the direction of effect. Second, DNA methylation levels were measured from buccal cells instead of hippocampal cells. Although we recognize that there may be differences in DNA methylation at specific CpG sites between hippocampal tissues and the cells available from a buccal swab, we note that buccal cells come from the same primordial lineage and that findings are similar from buccal and brain specimens used in studies of psychiatric traits (Smith et al., 2015). Third, the sample size for this study was small (*n* = 23), and variability within-participant significantly influenced the data. Larger sample size would have reduced this variability, reducing the probability of false negatives.

## Conclusion

In summary, epigenetic modifications of *NR3C1* may influence the ANS such that increased methylation of *NR3C1* exon 1_F_ at CpG sites 12 and 13 may be associated with increased activation of parasympathetic pathways as represented by increased HRV. This association provides a new channel of research when examining *NR3C1* methylation and stress response. Further in-depth exploration of the relationship between *NR3C1* methylation and ANS response is required to examine the mechanism through which the two measures are related.

## Data Availability Statement

The datasets for this study will not be made publicly available because the datasets have not been de-identified and were collected under a consent form that does not allow for sharing of the data outside of this study.

## Ethics Statement

This study was carried out in accordance with the recommendations of Women and Infants Hospital of Rhode Island Institutional review Board with written informed consent from all subjects. The protocol was approved by the Women and Infants Hospital of Rhode Island Institutional Review Board.

## Author Contributions

GA contributed to the conception of the manuscript, analyzed and interpreted the data, drafted the manuscript, circulated for review and revised the manuscript. EC conceptualized and designed the study, supervised data collection, and reviewed and revised the manuscript. JP conceptualized and designed the study, supervised the collection and prepared the biological samples for molecular analyses, reviewed and revised the manuscript. SS conceptualized and designed the study, coded the heart rate variables to synchronize with behavior and timing of paradigm episodes, reviewed and revised the manuscript. HT coded the heart rate variables to synchronize with behavior and timing of paradigm episodes, reviewed and revised the manuscript. LD analyzed and interpreted the data, reviewed and revised the manuscript. CM conceptualized and designed the study, conducted the molecular analyses, reviewed and revised the manuscript. BL and ET conceptualized and designed the study, reviewed and revised the manuscript.

## Conflict of Interest

The authors declare that the research was conducted in the absence of any commercial or financial relationships that could be construed as a potential conflict of interest.
